# Enhancing epigenetic aging clocks in cetaceans: accurate age estimations in small endangered delphinids, killer whales, pilot whales, belugas, humpbacks, and bowhead whales

**DOI:** 10.1038/s41598-025-86705-5

**Published:** 2025-02-03

**Authors:** Joseph A. Zoller, Ake T. Lu, Amin Haghani, Steve Horvath, Todd Robeck

**Affiliations:** 1 Department of Biostatistics, Fielding School of Public Health, University of California, Los Angeles, Los Angeles, CA USA; 2 Altos Labs, San Diego, CA USA; 3 SeaWorld Parks, 7007 SeaWorld Drive, Orlando, FL USA

**Keywords:** Epigenetic clock, DNA methylation, Cetacea, Beluga, Common bottlenose dolphin, Humpback whale, Killer whale, Bowhead whale, Methylation analysis, Machine learning

## Abstract

This study presents refined epigenetic clocks for cetaceans, building on previous research that estimated ages in several species from bottlenose dolphins to bowhead and humpback whales using cytosine methylation levels. We combined publicly available data (generated on the HorvathMammalMethylChip40 platform) from skin (n = 805) and blood (n = 286) samples across 13 cetacean species, aged 0 to 139 years. By combining methylation data from different sources, we enhanced our sample size, thereby strengthening the statistical validity of our clocks. We used elastic net regression with leave one sample out (LOO) and leave one species out (LOSO) cross validation to produce highly accurate blood only (Median Absolute Error [MAE] = 1.64 years, r = 0.96), skin only (MAE = 2.32 years, r = 0.94) and blood and skin multi-tissue (MAE = 2.24 years, r = 0.94) clocks. In addition, the LOSO blood and skin (MAE = 5.6 years, repeated measures r = 0.83), skin only (MAE = 6.22 years, repeated measures r = 0.81), and blood only (MAE = 4.11 years, repeated measures r = 0.95) clock analysis demonstrated relatively high correlation toward cetacean species not included within this current data set and provide evidence for a broader application of this model. Our results introduce a multi-species, two-tissue clock for broader applicability across cetaceans, alongside single-tissue multi-species clocks for blood and skin, which allow for more detailed aging analysis depending on the availability of samples. In addition, we developed species-specific clocks for enhanced precision, resulting in four blood-specific clocks and eight skin-specific clocks for individual species; all improving upon existing accuracy estimates for previously published species-specific clocks. By pooling methylation data from various studies, we increased our sample size, significantly enhancing the statistical power for building accurate clocks. These new epigenetic age estimators for cetaceans provide more accurate tools for aiding in conservation efforts of endangered cetaceans.

## Introduction

In the dynamic field of epigenetic aging clocks, the advent of the mammalian methylation array platform has revolutionized our ability to estimate the ages of mammals^1^. Our collaborative efforts, along with those of other researchers, have been instrumental in utilizing cytosine methylation levels, derived from these arrays, to create precise epigenetic age estimators for hundreds of mammalian species^2^. Notably, these methods have been applied successfully to a wide range of cetacean species, including multiple small delphinid species (e.g., bottlenose dolphins, pacific white-sided dolphins, common dolphin), beluga whales, killer whales, pilot whales, bowhead whales, and humpback whales, using both blood and skin samples. The application of these clocks, particularly in wild cetacean population management, offers a novel approach to understanding and improving the health and biology of these marine populations.

Traditionally, cetacean population monitoring has relied heavily on tooth growth layer identification, the identification of individual animals through photo identification, or unique morphological characteristics, all methods that are limited in scope or require extensive resources. However, the development of cetacean aging clocks has been significantly aided by cetaceans in aquaria, whose known ages provide crucial control data. These animals have been pivotal for clock development in various cetacean species and have opened the door for application towards the few localized populations of animals in the wild with known ages^3–8^.

While health assessments and associated blood sample collection from individuals within wild populations of cetaceans have become more common, the majority of biologic data collected from cetaceans, especially large odontocetes and baleen whales, is typically determined from the analysis of blubber and epidermal biopsies. Therefore, an accurate aging clock based on skin samples would have broad application across multiple taxa. In addition, improving the accuracy of these clocks across the entire age range of species may allow for the detection of abnormal biological age deviations of individuals or entire populations that have been subjected to unusual and ecological events. Detection of a difference between chronological age and biological age is the primary focus of human longevity research and has spawned a new industry dedicated toward defining and then improving these metrics.

Accurate chronological age estimation is important for endangered species populations that are facing extinction^9^. Our study aimed to further refine epigenetic clocks, enhancing their accuracy to estimate age based on blood and skin samples across all cetacean species. The study was inspired by universal pan-mammalian clocks, which allow one to estimate age across a multitude of mammalian species, unaffected by intra-species variation in lifespan^2^. This is exemplified in studies like those on dogs, where age estimates remained accurate despite considerable variations in life expectancy across breeds^10,11^.

While multi-species clocks are often less accurate than species-specific clocks, our approach overcomes this by leveraging large datasets collected from several studies, resulting in highly accurate cetacean clocks. Similar to the justification used for recent clock published for “long lived cetaceans”^8^, we hypothesize that the inclusion of a large number of species with wide age ranges (0 to 100 years) will reduce any age- related decreases in accuracy that many species-specific clocks have experienced. Therefore, we believe this unique combination of large sample numbers and a wide age range will improve the overall accuracy across the lifespan of the majority of species and allow for improved species-specific age estimations. This article presents a new multi-species cetacean clocks and related software, designed to surpass the accuracy and scope of previous epigenetic aging clocks, thereby contributing significantly to the fields of marine biology and cetacean conservation.

## Results

### Cetacean species methylation data

To establish epigenetic DNA methylation clocks for cetacean mammals, we used an array platform that quantifies genomic cytosine methylation at sufficient sequencing depth^1^. This mammalian methylation array platform profiles individual CpGs in highly conserved stretches of DNA in mammals. By design, the mammalian methylation array facilitates comparative studies across mammalian species (including humans) due to its very high sequencing depth (over thousand-fold) in highly-conserved CpGs in mammals. This Infinium array measures up to 36 k CpGs per species that are well conserved across many mammalian species. It features a probe set that can tolerate specific cross-species mutations. The array was previously annotated in over 200 species and reports CpG island status and chromatin states.

### Epigenetic clocks

**Fig. 1:**
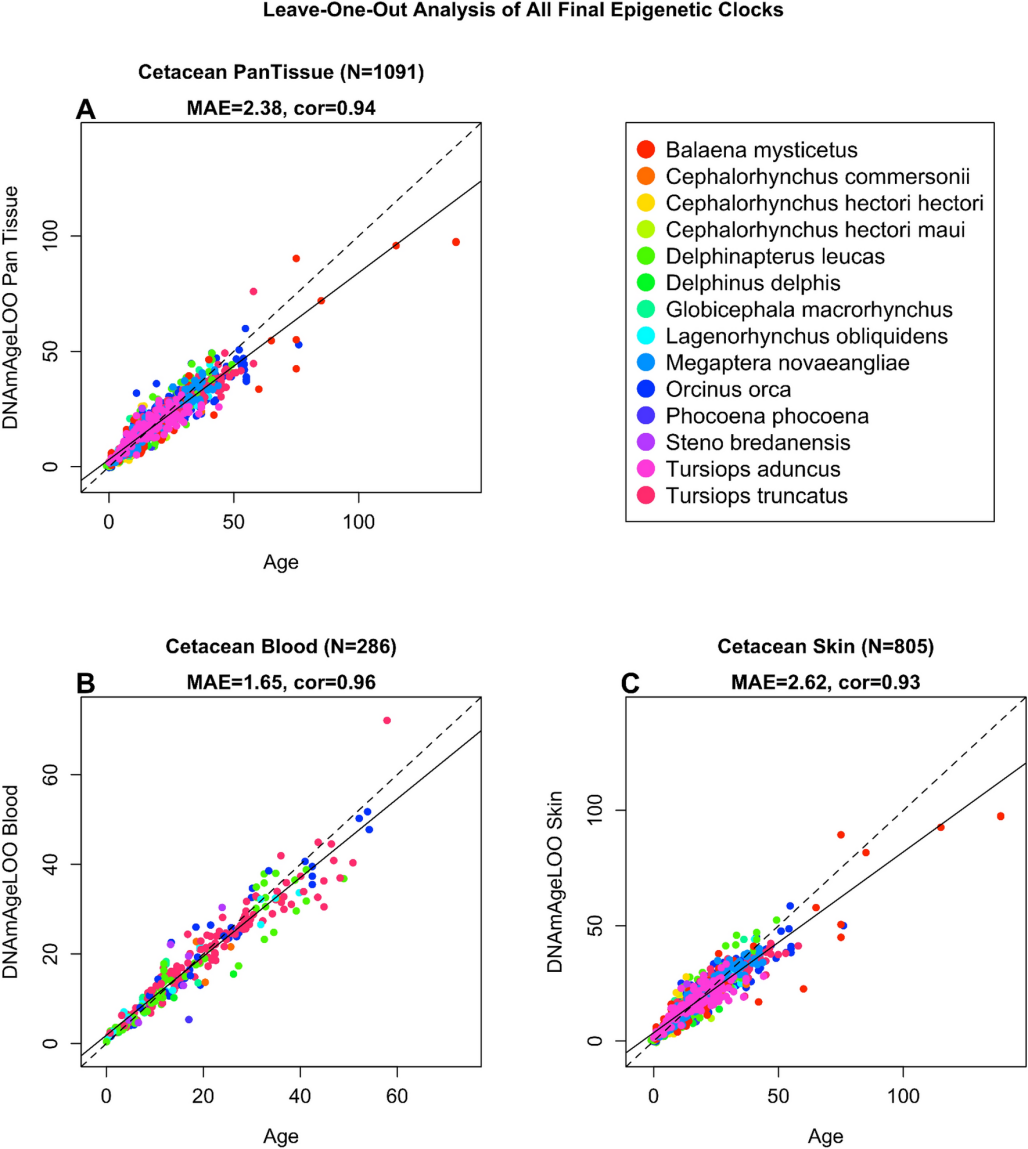
Cross-validation study of epigenetic clocks for cetaceans. Three epigenetic clocks that were trained on cetacean tissues: (**A**) all tissues, (**B**) blood, (**C**) skin. Dots are colored by species. “LOO” sample denotes the leave-one-out cross validation estimates of DNA methylation age (DNAm, y-axis, in units of years). Each panel illustrates a linear regression (black dashed line), a diagonal line (y = x), sample size (N), Pearson correlation (cor) between DNAm and estimated age across all samples, and median value of the median absolute error (MAE) for residuals across all samples.

For the construction of the multi-species, two-tissue cetacean clock, we utilized DNAm data previously generated with the HorvathMammalMethylChip40. This data comprised 1091 samples representing blood (n = 286) and skin (n = 805) from 13 different cetacean species, with individuals aged between 0 to 139 years. Additionally, we developed other cetacean clocks that can be categorized along two dimensions: species and tissue type. We stratified samples by tissue, creating two single-tissue, multi-species cetacean clocks specifically for blood and for skin. Our multi-tissue, multi-species clock selected 288 CpGs, which, after cross-validated using in Leave One Sample Out Cross Validation (LOOCV), resulted in a highly accurate epigenetic clock modal with a correlation coefficient (r) of 0.94 and a median absolute residual error (MAE) of DNAm age—observed age of 2.38 years (Fig. 1A). The two single-tissue, multi-species clocks produced cross validated accuracy values for blood of MAE = 1.65 years, r = 0.96, and for skin of MAE = 2.62 years, r = 0.93 (Fig. 1B,C). In addition, the high accuracy according to Leave One Species Out Cross Validation (LOSOCV) results from the multi-tissue clock (repeated measures r = 0.81, median MAE = 5.57 years [Supplementary Fig. 1A]); blood clock (repeated measures r = 0.95, median MAE = 4.60 years [Supplementary Fig. 1B]) and skin clock (repeated measures r = 0.79, median MAE = 6.81 years [Supplementary Fig. 1C]) indicate that these clocks are largely applicable to any cetacean species that were not part of the original training set.

**Fig. 2:**
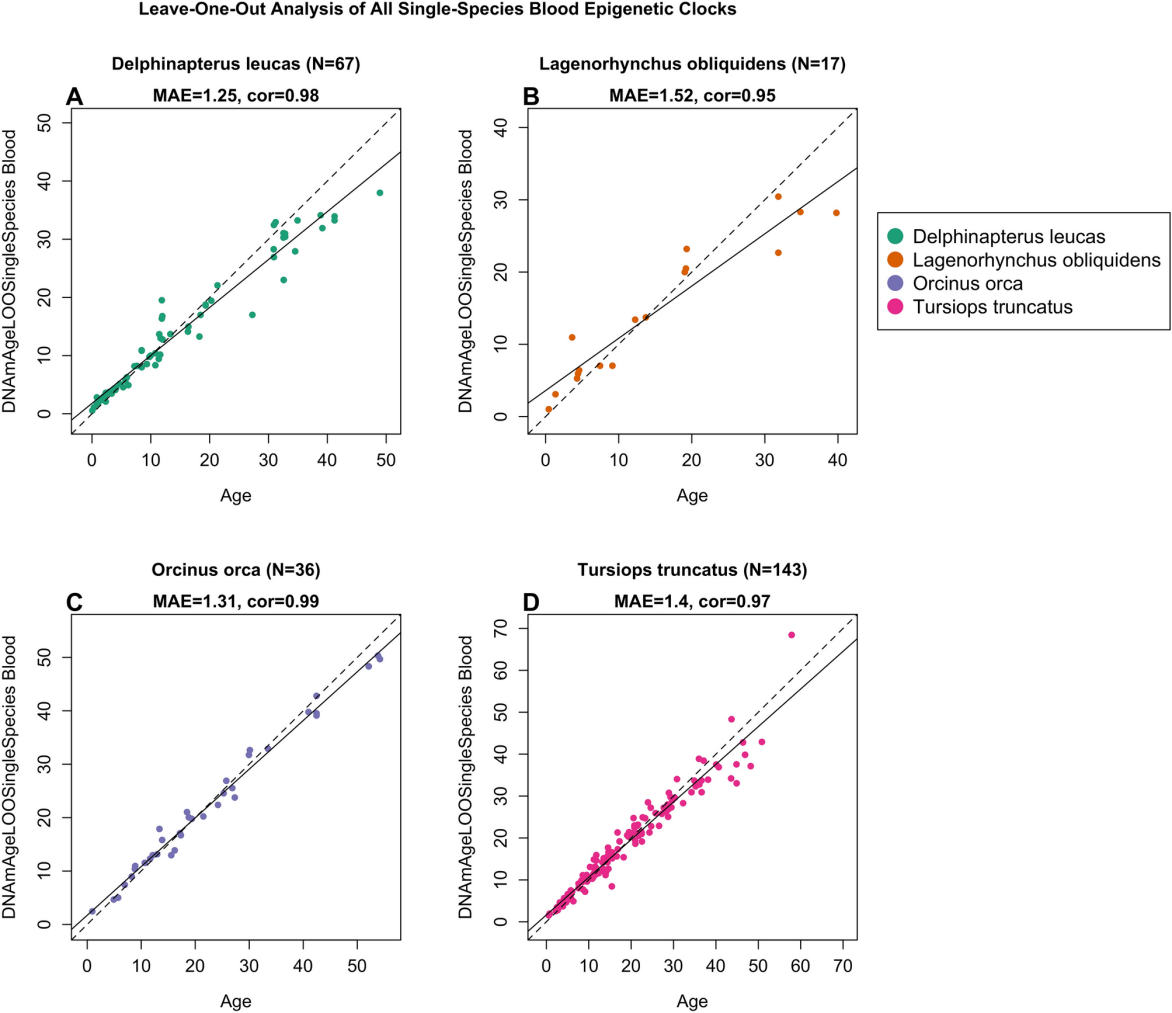
Cross-validation study of single-species epigenetic clocks for cetaceans for blood. Four epigenetic clocks that were trained on cetacean blood samples from individual species: (**A**) beluga whale, (**B**) pacific white-sided dolphin, (**C**) killer whale, (**D**) common bottlenose dolphin. Dots are colored by species. “LOO” sample denotes the leave-one-out cross validation estimates of DNA methylation age (DNAm, y-axis, in units of years). Each panel illustrates a linear regression (black dashed line), a diagonal line (y = x), sample size (N), Pearson correlation (cor) between DNAm and estimated age across all samples, and median value of the median absolute error (MAE) for residuals across all samples.

**Fig. 3:**
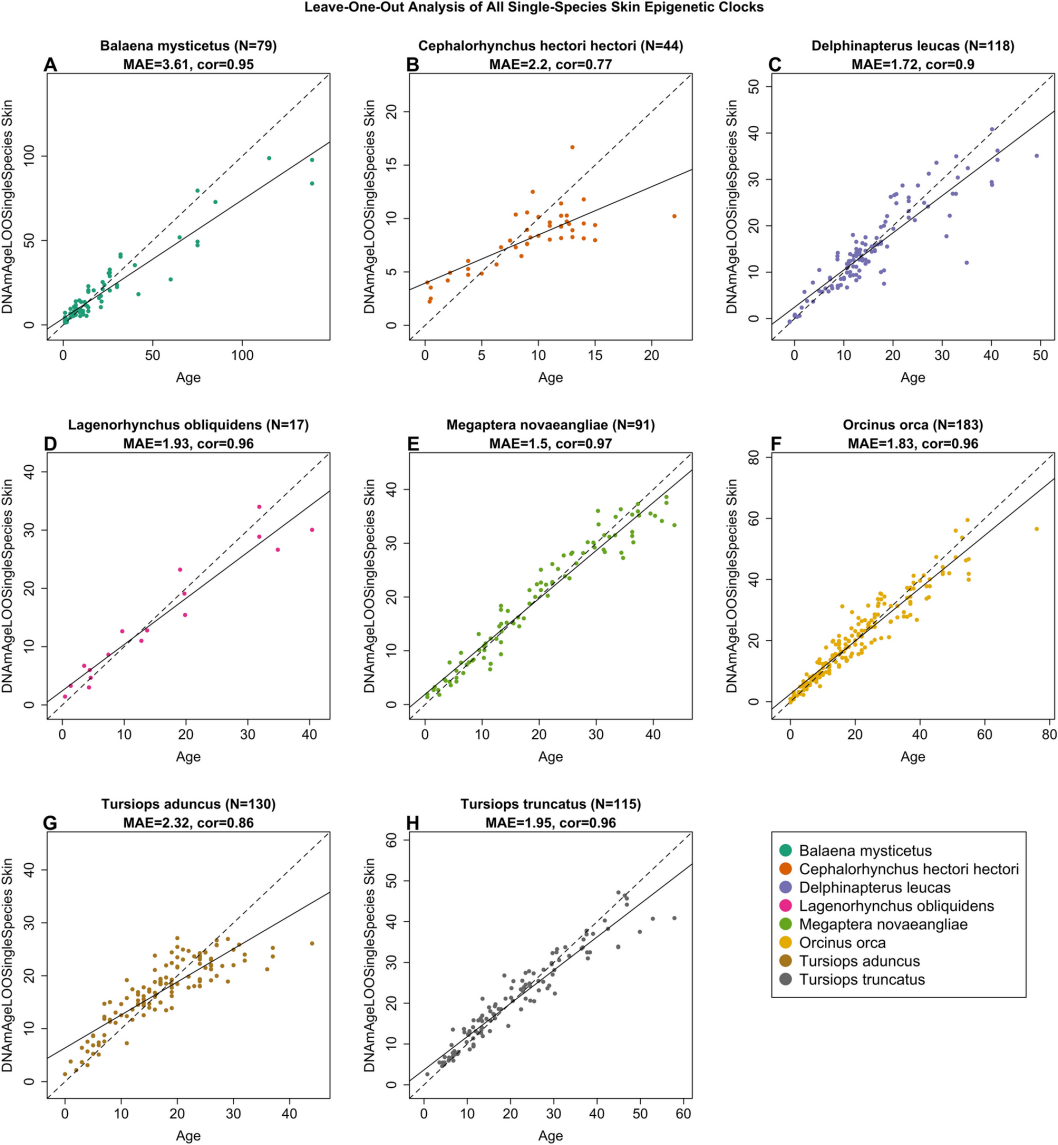
Cross-validation study of single-species epigenetic clocks for cetaceans for skin. Eight epigenetic clocks that were trained on cetacean skin samples from individual species: (**A**) bowhead whale, (**B**) Hector’s dolphin, (**C**) beluga whale, (**D**) pacific white-sided dolphin, (**E**) humpback whale, (**F**) killer whale, (**G**) Indo-pacific bottlenose dolphin, (**H**) common bottlenose dolphin. Dots are colored by species. “LOO” sample denotes the leave-one-out cross validation estimates of DNA methylation age (DNAm, y-axis, in units of years). Each panel illustrates a linear regression (black dashed line), a diagonal line (y = x), sample size (N), Pearson correlation (cor) between DNAm and estimated age across all samples, and median value of the median absolute error (MAE) for residuals across all samples.

Furthermore, we created species-specific clocks, which were trained on either all blood samples or all skin samples from a single species. This process resulted in four distinct blood-based clocks and eight distinct skin-based species-specific clocks. It is important to note that these species-specific clocks are designed to be applicable to a single cetacean species and a single tissue type and are not expected to be valid when applied to other cetacean species or other tissue types. Within these four blood clocks, the LOOCV MAE ranged from 1.25 years (beluga, r = 0.98) to 1.52 years (pacific white-sided dolphin, r = 0.95) (Fig. 2), and within the eight skin clocks they ranged from 1.50 years (humpback, r = 0.97) to 3.61 years (bowhead, r = 0.95) (Fig. 3). The results from these clocks perform favorably when compared to previously published species-specific clocks (Table 1), and highlight both the conserved nature of age-related CpGs, and the importance of sample size toward clock accuracy.

**Table 1:** Accuracy (mean absolute error [in years], Pearson’s correlation) comparisons of published DNA methylation aging clocks within cetacean species for skin samples, using leave-one-sample-out cross-validation.

Common name	Cetacean^ǂ^	Odontocete^4^	Species-specific
Beluga	1.72, 0.90	2.72, 0.94	2.9, 0.86* (Bors et al., 2021)^27^
Bowhead whale	3.61, 0.95	–	3.73, 0.95 (Parsons et al., 2023)^8^
Common bottlenose dolphin	1.95, 0.96	4.8, 0.91	2.53, 0.95 (Robeck et al., 2021a)^3^
Hector’s dolphin	2.20, 0.77	N/A	2.08, 0.87* (Hernandez et al., 2023)^28^
Humpback whale	1.50, 0.97	–	2.21, 0.96 (Horvath et al., 2022)^6^
Indo-Pacific bottlenose dolphin	2.32, 0.86	3.5, 0.76	2.1, 0.86 (Peters et al., 2023)^7^
Killer whale	1.83, 0.96	3.1, 0.89	2.26, 0.96 (Parsons et al., 2023)^8^
Pacific white-sided dolphin	2.14, 0.92	1.69, 0.97	N/A

^ǂ^Results from this research (skin-specific clock), *Calculated from R^2^.

To ensure unbiased estimates of the epigenetic clocks, we employed leave-one-out (LOO) cross-validation on the training data. This cross-validation study provided unbiased estimates of the age correlation R (defined as the Pearson correlation between the DNAm age estimate and chronological age) and the median absolute error (MAE), which measures the deviation between predicted and observed ages (in chronological years). The results indicate that the pan-clock is highly accurate in estimating ages of both blood and skin samples across different animals (Supplementary Fig. 2). Similarly, the blood-clock and skin-clock show high accuracy in age estimation of samples from various animals (Supplementary Fig. 3, and Supplementary Fig. 4, respectively).

### Meta epigenome-wide association study of age across species

Cetacean clocks, developed using penalized regression models, are composed exclusively of CpG sites that are most predictive of age, resulting in the exclusion of many other age-related CpG sites from the final models. In contrast, epigenome-wide association studies (EWAS) examine each CpG site individually, correlating each with age. To identify CpG sites that consistently relate to age across different cetacean species, we employed a meta-analysis approach. Specifically, we conducted a two-stage meta-analysis EWAS across multiple species and tissues in cetaceans. The resulting CpG sites are consistently associated with age across different tissues and cetacean species.

**Fig. 4:**
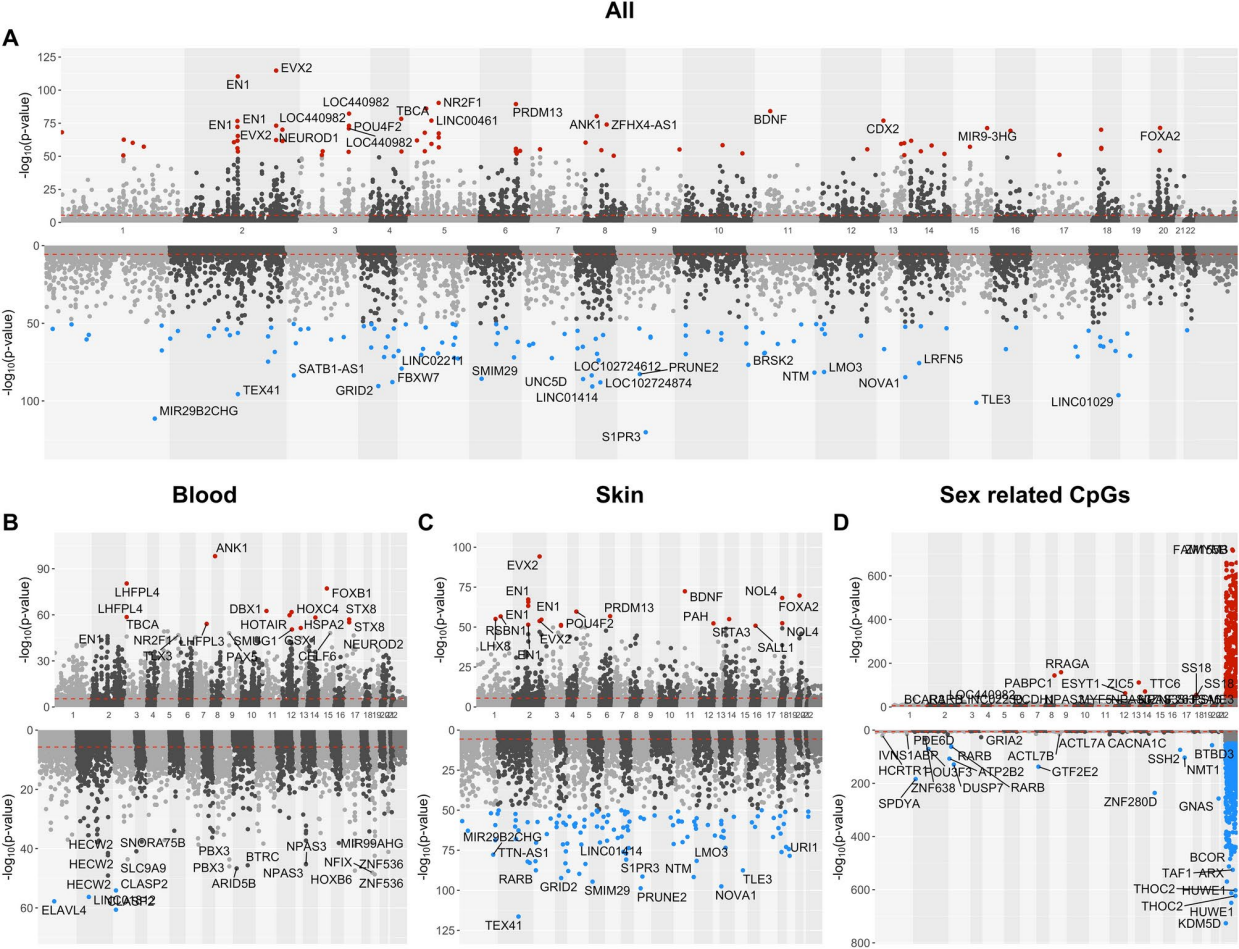
Epigenome-wide association study (EWAS) of age and sex across cetacean species. Each panel presents the results of the EWAS across cetacean species, using the same data as in Fig. 1. The objective of the meta-analysis was to identify individual CpGs with significant positive (the upper part) or negative correlations with age (the lower part in panels **A**–**C**) or sex (panel **D**). (**A**) Meta-analysis of age across both blood and skin tissues using a two-step approach. Step 1 involved conducting EWAS for age across different species-tissue categories and applying Stouffer’s method to generate estimates at the species level. Step 2 then combined these species-specific EWAS results to generate meta-estimates based on Stouffer’s method. (**B**) Meta-analysis EWAS of age in blood samples only. (**C**) Meta-analysis EWAS of age in skin samples only. (**D**) Meta-analysis EWAS of sex, upper/lower panels indicating increased/decreased methylation in females. This analysis followed a two-step approach similar to that in Panel (**A**). For each species-tissue category, the EWAS for sex-associated CpGs was adjusted for age using a multivariate linear regression model, lm(CpG ~ Age + Sex), with Age included as a covariate. As expected, associations are enriched on the X chromosome. The red dashed lines represent an uncorrected two-sided nominal significance threshold for Bonferroni correction, approximately 2.5 × 10^–6^.

The top 50 positively and negatively age-related CpG sites surpass stringent unadjusted significance thresholds of α = 5.3 × 10^–56^ and α = 3.3 × 10^–56^, respectively (gene annotations shown in Fig. 4A and Supplementary Data 1). Among the most significant positively correlated variants were cg09227065 on chromosome 2 in *EVX2* (Meta P = 1.7 × 10^–115^), cg13400013 on chromosome 2 in *EN1* (Meta P = 4.3 × 10^–111^), and cg27201382 on chromosome 11 in BDNF (Meta P = 7.7 × 10^–85^). Conversely, for negatively correlated variants, the most significant included cg17856858 on chromosome 9 in *S1PR3* (Meta P = 6.8 × 10^–121^), cg26931400 on chromosome 15 in *TLE3* (Meta P = 6.9 × 10^–102^), and cg01893274 on chromosome 2 in *TEX41* (Meta P = 2.2 × 10^–96^). Several of these genes, including *EVX2*, *EN1*, *BDNF*, *TLE3*, and *S1PR3*, are implicated in developmental processes.

In our blood EWAS, the top significant age-related genes with positive correlations include *LHPL4*, *LHPL3*, *FOXB1*, *HOXC4*, and others. Conversely, genes such as *CLASP2*, *ELAVL4*, and others showed significant negative correlations (Fig. 4B). Notably, both cg12841266 (Meta P = 2.7 × 10^–59^) and cg11084334 (Meta P = 4.0 × 10^–81^) on chromosome 3 in *LHPL4* are the top two age-related CpGs previously identified in our cross-species EWAS of age across various eutherian mammals^2^.

In our skin EWAS, several transcription factors emerged as top significant age-related genes with positive correlations, including *LHX8*, *TFAP2D*, and *PHOX2B*. Novel genes showing negative age correlations include *RARB*, involved in retinoic acid signaling, the interleukin gene *IL20RB*, and *PRDM13*, a transcription factor implicated in neural development, among others (Fig. 4C).

As expected, the top CpGs from our sex-based EWAS were predominantly located on the sex chromosomes (Fig. 4D). These CpGs were identified through regression analyses adjusted for age effects. In the autosomes, several key genes involved in well-established biological pathways or functional sets were identified. For example, CpGs exhibiting higher methylation levels in female cetaceans were linked to genes such as the mTOR-related *RRAGA* on chromosome 9 (cg04565674, Meta P = 1.0 × 10^–159^) and the breast cancer-related gene *BCAR3* on chromosome 1 (cg06678289, Meta P = 4.6 × 10^–36^), among others (upper panel in Fig. 4D). Conversely, for CpGs exhibiting lower methylation levels in female cetaceans, notable genes included *GNAS* on chromosome 20, involved in hormone receptor signaling (cg26452915, Meta P = 3.68 × 10^–258^); *ATP2B2* on chromosome 3, involved in calcium channel activity (cg24358591, Meta P = 5.5 × 10^–108^); and *POU3F3* on chromosome 2 (cg16767916, Meta P = 9.6 × 10^–72^), among others (lower panel in Fig. 4D).

### Chromatin state analysis of EWAS of age

To understand the epigenetic context of age-related CpG sites, we utilized a comprehensive universal chromatin state annotation of the human genome. This resource, derived from 1032 experiments mapping 32 chromatin marks across over 100 human cell and tissue types^12^, enabled us to overlay the positions of the top 1000 positively and top 1000 negatively age-related CpG sites. We found that positively age-related CpG sites were significantly enriched in states associated with polycomb repressive complex 2 (PRC2)-binding sites (states BivProm1, BivProm2, and ReprPC1). These CpG sites localized to PRC2-binding sites, as defined by embryonic ectoderm development (EED), enhancer of zeste 2 (EZH2), and SUZ12 binding.

**Fig. 5:**
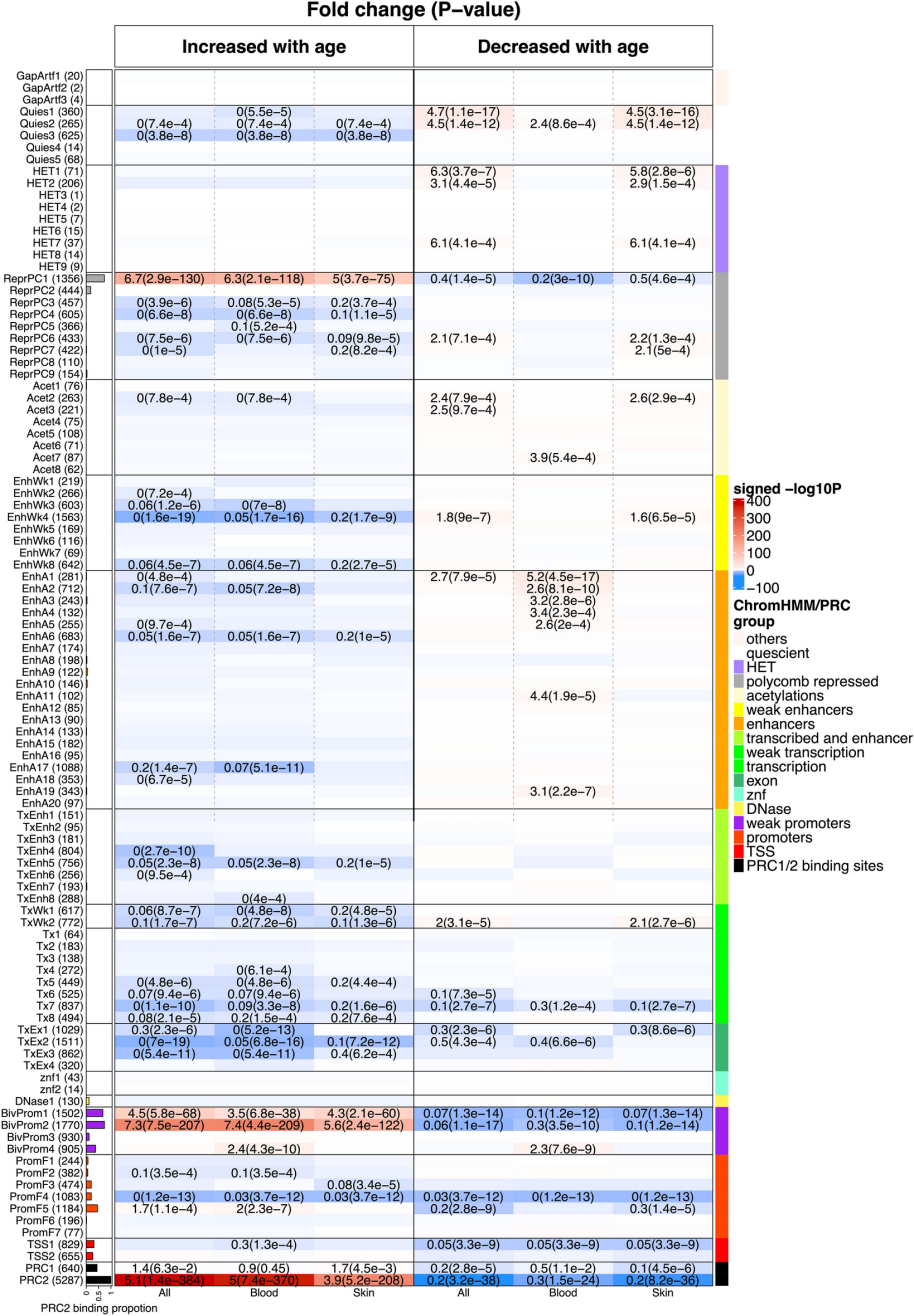
Chromatin state analysis of EWAS of age. The rows correspond to universal chromatin states from Vu and Ernst 2022. The columns (x-axis labels) correspond to the EWAS results of age reported in Fig. 4. The entries of the table report the enrichment scores. For chromatin state annotation, the most enriched states/groups are still BivProm2, BivProm1 and PRC2 based on top 1000 CpGs with positive age correlation.

This PRC2 enrichment was observed across both tissue types collectively (fold change [FC] = 5.1, hypergeometric P = 1.4 × 10^−384^) and when analyzed individually: blood (FC = 5.0, P = 7.4 × 10^−370^) and skin (FC = 3.9, P = 5.2 × 10^−208^, Fig. 5 and Supplementary Data 2).

PRC2, a transcriptional repressor complex, is a key contributor to H3K27 methylation, a chromatin modification linked to transcriptional repression. PRC2-mediated histone 3 lysine 27 (H3K27) methylation is crucial for establishing bivalent promoters, which feature both H3K27 trimethylation (H3K27me3) and histone 3 lysine 4 trimethylation (H3K4me3). Thus, it is consistent that positively age-related CpG sites are enriched in bivalent promoter states (e.g., BivProm1 and BivProm2, see the first column of Fig. 5). They are particularly prevalent in the bivalent state associated with more H3K27me3 than H3K4me3 (BivProm2), compared to BivProm1, which has more balanced levels of these histone modifications. These mammalian results mirror those from our pan-mammalian studies (Lu, 2023, Universal DNA methylation age across mammalian tissues), where tissue-independent age-related methylation gain was characterized by cytosines located in PRC2-binding sites and bivalent chromatin domains. Similar findings have been reported in previous studies focused on specific species, such as humans^13,14^.

Age-related loss of methylation was observed in CpG sites located in select heterochromatin states (HET1, HET2), marked by histone 3 lysine 9 trimethylation (H3K9me3), or in inactive chromatin states (Quies1, Quies2). We reported similar results for proliferative tissues (including blood and skin) in our pan-mammalian studies^2^.

### Functional enrichment analysis of age-related CpG sites

**Fig. 6:**
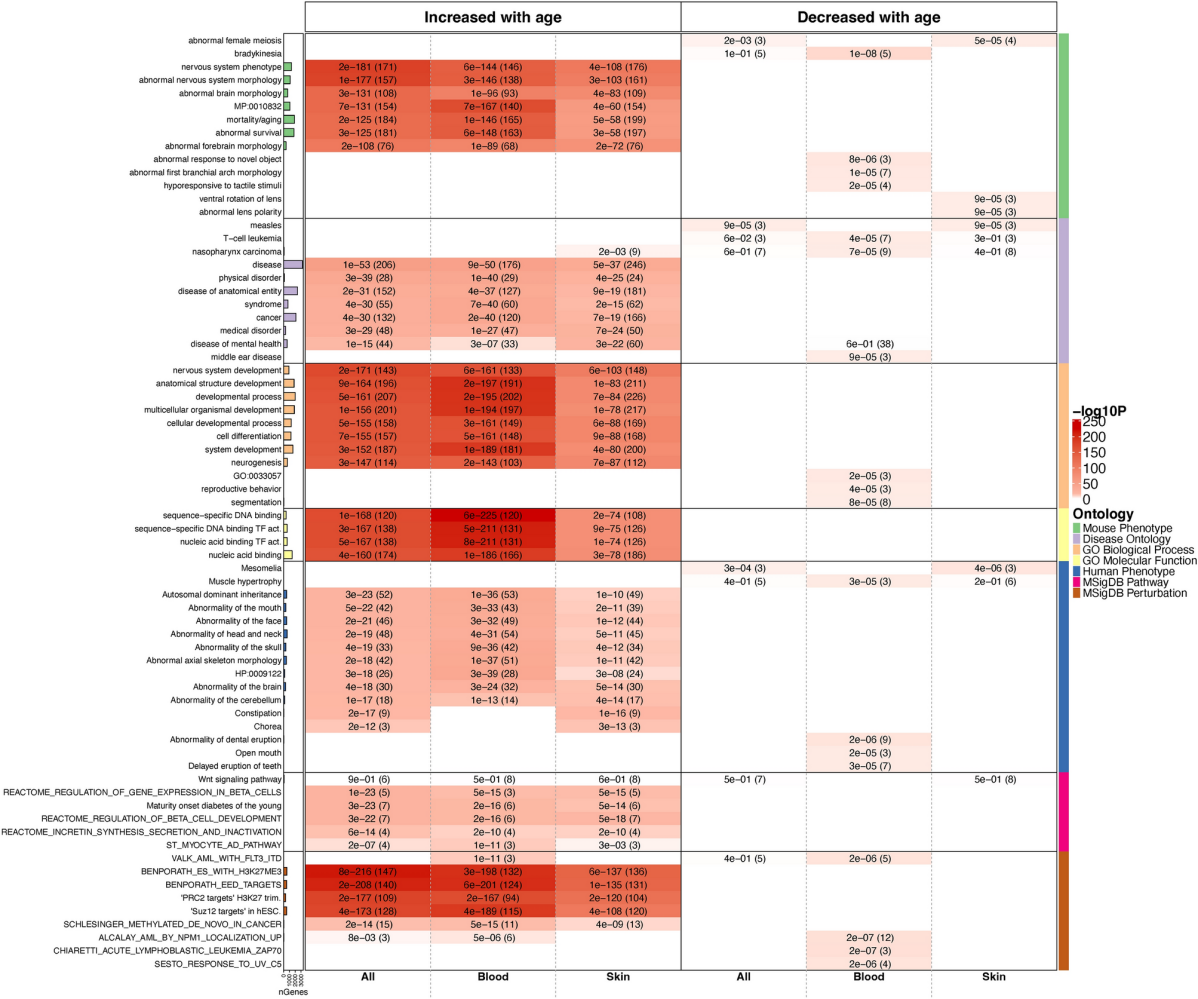
Genomic-Region Based GREAT Functional Enrichment. The analysis is based on a foreground/background hypergeometric test, with the background consisting of genomic regions from 37 k mammalian CpGs and the foreground comprising genomic regions from the top CpGs identified in our meta-EWAS of age. The left and right panels present the enrichment analysis for the top 1000 CpGs that increased or decreased with age, respectively. The columns along the x-axis represent results from the EWAS across all tissues, blood, and skin types. The rows on the y-axis are organized by different ontologies. The bar plots in the leftmost column indicate the size of each annotation set (number of genes), while the color band in the rightmost column depicts seven distinct ontologies. Each cell contains the unadjusted hypergeometric p-value and the corresponding number of overlapping genes. Description for the annotation sets can be found in Supplementary Data 2.

We used the Genomic Regions Enrichment of Annotations Tool (GREAT) to annotate the potential function of cis regulatory regions of age-related CpG sites^15^. We sought to identify biological processes and pathways potentially associated with the top 1000 positively and negatively age-related CpG sites (Fig. 6). To avoid array-design bias, we used mammalian array CpG sites as a background set in our hypergeometric enrichment test.

Analysis of CpG sites positively correlated across all tissues revealed ‘nervous system development’ as a highly significant gene ontology (GO) term (P = 2.0 × 10^–171^). This term was consistent across blood (P = 6.0 × 10^–161^) and skin (P = 6.0 × 10^–103^). Other significant GO terms included ‘developmental process’, ‘regulation of RNA metabolic process’, ‘nucleic acid-binding TF activity’, ‘pattern specification’ and ‘anatomical structure development’ (Fig. 6 and Supplementary Data 3). The GREAT analysis also indicated that a significant proportion of the top 1,000 positively age-related CpG sites are located in PRC2 target sites: P = 8.0 × 10^–216^ for all tissue types, P = 3.0 × 10^–198^ for blood, and P = 6.0 × 10^–137^, which was also true for individual core PRC2 subunits (SUZ12, EED or EZH2; Fig. 6).

### Transcription factor binding site enrichment analysis

We annotated CpG sites with binding sites for 274 transcription factors (TFs) using data from the JASPAR database, as implemented in the R LOLA package. TF binding sites were identified through ChIP-seq, SELEX, or HT-SELEX methods and were sourced from the JASPAR database (https://jaspar.elixir.no/enrichment/). To assess overlap with the binding sites, we defined a buffer region of 200 base pairs (bp) around the CpG sites.

**Fig. 7:**
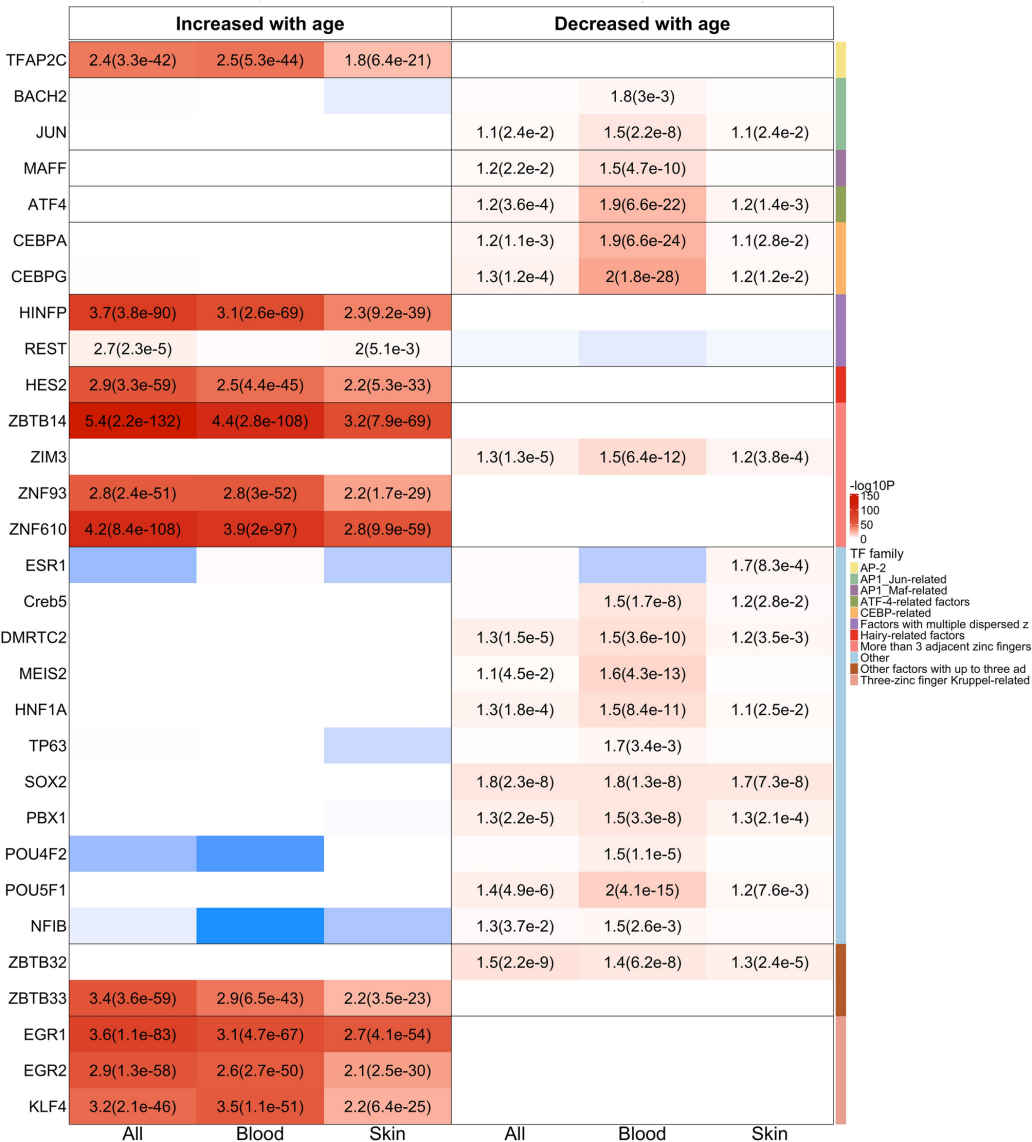
Genomic-region based transcription factor binding site enrichment analysis. The heatmap displays transcription factor (TF) binding site enrichment for age-associated CpGs, evaluated against a background surrounding 37 k mammalian CpGs with a 200 bp buffer. The left and right panels present the enrichment analysis for the top 1000 CpGs that increased or decreased with age, respectively. The columns along the x-axis represent results from EWAS across all tissues, blood, and skin types. Rows are organized by TF genes grouped into different families, with color bands in the rightmost column indicating these families. Each cell shows an odds ratio (unadj. hypergeometric p-value) where p < 0.05.

Our analysis revealed pronounced enrichments for CpGs that gain methylation with age (Fig. 7 and Supplementary Data 4). The most significantly enriched TF, ZBTB14, a member of the ZBTB family, demonstrated strong associations: overall odds ratio (OR) = 5.4 with an unadjusted hypergeometric P-value of 2.2 × 10^–132^; OR = 4.4 (P = 2.8 × 10^–108^) in blood tissue; and OR = 3.2 (P = 7.9 × 10^–69^) in skin tissue. Other notable TF enrichments observed across all tissue types include HINFP, which regulates histone genes (OR = 3.7, P = 3.8 × 10^–90^), KLF4 (OR = 3.2, P = 2.1 × 10–46), and REST (OR = 2.7, P = 2.3 × 10^–5^). The enrichment of REST is consistent with our previous epigenome-wide association study (EWAS) findings on aging across various mammalian species^2^.

In contrast, enrichments associated with CpGs that lose methylation with age were relatively weak, with stronger enrichments observed in blood compared to skin tissue. In the blood EWAS of age, the most significant enrichments were observed for the CEBP family genes, particularly CEBPG (OR = 2, P = 1.8 × 10–^28^), followed by the Yamanaka factor OCT4 (OR = 2, P = 4.1 × 10^–15^). Additional noteworthy TF enrichments included another Yamanaka factor (SOX2), as well as AP-1 related genes such as JUN, and the ATF family gene ATF4. Enrichments also involved other significant genes within the AP-1 and ATF-4 related families.

## Discussion

The realm of epigenetic research has witnessed the publication of numerous clocks tailored for various mammalian species; cetaceans included. The distinguishing feature of our study, setting it apart from its predecessors, is predominantly technical. By harnessing the power of a considerably more extensive dataset than what was utilized in earlier research, we have paved the way for the creation of clocks that are anticipated to surpass the performance of those previously established.

However, it’s crucial to acknowledge the potential pitfalls associated with epigenetic clocks. One of the primary challenges is the likelihood of a systematic offset when applied to new data. Such discrepancies can inadvertently skew results, leading to inaccuracies in age estimation. To counteract this, a pragmatic approach involves the incorporation of animals with a verified age into a test dataset. By doing so, it becomes feasible to estimate and account for this offset, ensuring the reliability and accuracy of the epigenetic clocks in real-world applications. As the field continues to evolve, such considerations will be paramount in refining and optimizing the utility of these innovative tools.

The large number of samples accumulated within this study set help provide for a level of accuracy which has yet to be reached for any species-specific, single-tissue DNAm aging clock. The difficulty in creating such a large data set of known age or approximately known age animals is obvious, and by combining previously published data sets into one training set we can overcome these limitations.

Most published species-specific aging clocks appear to have increased MAE with age, this is most likely due to the inaccuracy of aging techniques used to identify the age of adult animals. The majority of delphinid species have been aged using growth layer groups (GLGs) found within teeth. While this method is considered relatively accurate for young animals, gross differences in age estimates are apparent once animals reach adulthood and beyond^16^. Within bottlenose dolphins, errors in age estimation for older animals have been documented as large as 37 years^16^. Therefore, epigenetic clocks, which rely heavily on estimated ages for animals to build a large training set, as opposed to known ages for animals, will naturally have large residual deviations with age if these age estimations were inaccurate. For baleen whales, robust, long-running, photo id programs of humpback whales have allowed accurate identification of individual whales prior to maturation and this accuracy resulted in our species-specific skin clock producing very accurate age predictions of less than two years (LOOCV, MAE 1.50 years, r = 0.97) for this species across its age range. Similar situations were also evident for wild killer whales where-bye individuals have been identified at an early age and then followed for up to 50+ years. The accuracy of photo id programs is evident from our results whereby we combined data from known age aquarium housed animals with those from known age photo IDs wild animals and found slightly improved age estimation accuracy (MAE 1.83 years, r = 0.96) as compared to a previous publication that relied exclusively on wild killer whales with known or estimated birth years (MAE 2.26 years, r = 0.96)^8^. Therefore, the importance of using known age animals which have been aged based on unequivocal means—e.g., observation of birth or allometrically during juvenile age, is paramount for the development of accurate epigenetic clocks.

The addition of two species, with known or estimated ages, and overlapping and extended age ranges to increase the accuracy across the entire lifespan was recently utilized for bowhead and killer whales^8^. By adding multiple species, many of whom are considered to be known age, we have furthered the accuracy by creating aging clocks by with uniform residual distribution until approximately the chronological age of 60 years, a point at which approaches the maximum predicted maximum longevity for all but the killer whale (70 to 80 years) and the bowhead (211 years)^17^.

The extreme accuracy of the blood clocks presented herein provide a gold standard for age estimation of cetaceans. The single species clocks, for both blood and skin, are typically more accurate for age estimation than multispecies clocks, and this was demonstrated herein by the improved MAE for the blood clocks for beluga (MAE 1.25 years, n = 67), bottlenose dolphin (MAE 1.40 years, n = 143), killer whale (MAE 1.31 years, n = 36), and pacific white-sided dolphin (MAE 1.52 years, n = 17) (Fig. 2) as compared to the cetacean multi species blood clock (MAE 1.65 years) (Fig. 1B). However, these differences are small and demonstrate the value of the cetacean multi species clocks for estimating age in species whereby sample numbers will always be small. The routine collection of blood samples is rare from most species in the wild unless they are involved in subsistence harvests or regular health assessments (e.g., beluga^18,19^). However, catch and release health assessments are becoming increasingly more common with nearshore cetaceans, as are stranded animal response teams for beached or entangled animals from multiple species, whereby blood samples are often collected, and a portion of these samples can now be used to accurately estimate the animal’s age.

Our meta-analysis of epigenome-wide association studies of age across multiple cetacean species and tissues has uncovered CpG sites consistently associated with aging in cetacean species. As expected, based on previous works, we found that positively age-related CpG sites are significantly enriched in genes involved in developmental processes, particularly nervous system development. These CpG sites are localized to polycomb repressive complex 2 (PRC2) binding sites and bivalent promoter regions.

Transcription factor binding site analysis revealed significant enrichment for TFs such as ZBTB14, HINFP, KLF4, and REST, highlighting their association with age-related cytosine methylation changes. These findings align with previous studies in humans and other mammals, emphasizing the conserved nature of these methylation patterns. Among these transcription factors, ZBTB14, a member of the ZBTB family, remains poorly characterized but is expressed across various blood cell types (*The Human Protein Atlas*). Notably, another ZBTB family member, ZBTB33, also exhibits age-related activity in murine cells^20^. KLF4 has been previously identified by Maity et al.^20^, where an age-associated decline in its activity was observed in murine monocytes and macrophages. This decline reduces differentiation activity and may shift macrophage polarization from an M1 pro-inflammatory program toward an M2 tumor-promoting program. Given that DNA methylation is critical for suppressing transposable elements, the role of HINFP is particularly noteworthy, as it functions as a guardian of the somatic genome by repressing these elements^21^. Finally, the enrichment of REST aligns with findings from our previous epigenome-wide association study (EWAS) on aging across multiple mammalian species^2^.

Conversely, age-related loss of methylation was associated with heterochromatin, inactive chromatin states, and, to some extent, enhancers. Transcription factor analysis implicated Activator Protein 1 (AP-1)-related transcription factors, consistent with findings from our pan-mammalian studies^2^.

Overall, our results provide both insights into the epigenetic underpinnings of aging in cetaceans and an accurate aging clock with broad application towards multiple cetacean species.

## Methods

### Study subjects

Previously published Methylation results from samples which had been collected from 13 different cetacean species from known age or approximately known age animals living in both aquariums and in the wild (Table 2). Animals were considered known age if either born under human care or observed as neonates in the wild. For estimated age animals, there included animals which were either collected or observed (and uniquely identified) prior to physical maturity and their age was based on either total length or the time from initial observation as a juvenile until physical or sexual maturity was obtained, a technique commonly known as allometric age estimation. Small wild delphinids were aged based on tooth growth layers (GlG) during health exams. However, due to the inaccuracy of GLG estimation in beluga, no wild animals were included which relied solely on GLG age estimations. Finally, the ages of several species were estimated based on other methods, for example, for the bowhead whale age estimations of adult animals was performed using aspartic acid racemization^22^.

**Table 2:** Summary of training data samples for multi-species and species-specific clocks, by tissue type and source.

Common name	Blood samples	Skin samples	Data source(s)
Beluga	67	118	Robeck et al. (2021b)^4^ Bors et al. (2021)^27^
Bowhead whale	0	79	Parsons et al. (2023)^8^
Commerson’s dolphin	4	4	Robeck et al. (2021b)^4^
Common bottlenose dolphin	143	115	Robeck et al. (2021a)^3^
Harbour porpoise	1	1	Robeck et al. (2021b)^4^
Hector’s dolphin	0	52	Hernandez et al. (2023)^28^
Humpback whale	0	91	Horvath et al. (2022)^6^ + 1 skin sample previously unpublished
Indo-Pacific bottlenose dolphin	0	130	Peters et al. (2023)^7^
Killer whale	36	183	Parsons et al. (2023)^8^
Pacific white-sided dolphin	17	17	Robeck et al. (2021b)^4^
Rough-toothed dolphin	6	6	Robeck et al. (2021b)^4^
Short-beaked common dolphin	3	3	Robeck et al. (2021b)^4^
Short-finned pilot whale	9	6	Robeck et al. (2021b)^4^
Total samples	286	805	
Grand total	1091		

### Cetacean tissue samples

No new blood or skin samples were collected for this study, we combined data from previously analyzed samples (n = 1091), representing 13 different cetaceans. Details for sample collection and permits required varied for each species and have been previously published (Table 2). The data represent a subset of the results compiled by the Mammalian Methylation Consortium^2,23^.

### DNA methylation data

All DNA methylation data were generated using the mammalian Infinium array “HorvathMammalMethylChip40”^1^. By design, the mammalian methylation array facilitates epigenetic studies across mammalian species (including humans) due to its very high coverage (over thousand-fold) of highly-conserved CpGs in mammals.

The Infinium method is based on sodium bisulfite conversion of DNA and microarray-based genotyping of CpG sites using Infinium bead technology with single-base resolution. The advantage of the microarray platform is that it is user-friendly, it can be multiplexed, and it exhibits good agreement with other platforms’ DNA methylation measures. Specifically, the Infinium beads bear a 23-base oligo address to locate them on the BeadChip, and a 50-base probe. Probe sequences are complementary to specific 50 base regions of bisulfite-converted genomic DNA. The 3′ end of the probe harbors the methylated CpG site to be monitored. After the probe is hybridized to bisulfite-treated test DNA, a single-base extension adds a fluorescently labeled ddNTP to the 3′ CpG site. This lets the C to T change caused by bisulfite conversion to be "genotyped." The fluorescent signal is then measured and processed.

Out of all CpGs on the mammalian array, 20,150 CpGs map to a large fraction of the cetacean species’ genomes^1^. However, when building all of the clocks for this study using only mappable CpGs, we find that overall performance of the clocks (via cross-validation analysis) became mildly or substantially worse, and age predictions in some species became very inaccurate within the cetacean multi species clocks. Therefore, we elected to use all CpGs on the mammalian array in the building of the clocks presented. Genome coordinate information can be downloaded from our GitHub page (https://github.com/shorvath/MammalianMethylationConsortium) and the supplementary information in^1^. The chip manifest file can be found at Gene Expression Omnibus (GEO) at NCBI as platform GPL28271. The SeSaMe normalization method was used to define beta values for each probe^24^.

### Penalized regression models

Details on the clocks (CpGs) and R software code are provided in the Supplement and in our R software package^25^.

Due to a bias of samples being overrepresented within younger age ranges (both from having increased numbers of young animals within each species and the majority of species having life expectancies of less than 50 years^17^, we log-like transformed the chronological age prior to analysis (see Supplementary Materials for details on age transformation). Penalized regression models were created with glmnet^26^. We investigated models produced by “elastic net” regression (alpha = 0.5). The optimal penalty parameters in all cases were determined automatically by using a tenfold internal cross-validation (cv.glmnet) on the training set. By definition, the alpha value for the elastic net regression was set to 0.5 (midpoint between Ridge and Lasso type regression) and was not optimized for model performance.

We performed two cross-validation schemes for arriving at unbiased (or at least less biased) estimates of the accuracy of the different DNAm based age estimators. First, for validation of model application across the 13 cetacean species used for model development, we used leave-one-out LOO cross-validation (LOOcv) in which one sample was left out of the regression, then predicted the age for the remaining samples and iterated this process over all samples. The second method was similar to LOO, and previously developed for the odontocete clock^4^, was identified as leave-one-species-out (LOSOcv) cross validation, whereby, instead of leaving one random individual sample out of the model, we randomly left one species out of the model training set, optimized the model and then predicted the age the “unknown species” compared against the observed age. The LOOcv reports Pearson’s correlation (r) between DNAm age estimations and chronological age observations as well as the median absolute error (MAE) for the absolute error of the residuals (actual error of predicted versus observed age). For the LOSOcv, we report the repeated measures correlation (treating the same species as repeated measures), as well as the median MAE across the species. This was done to reduce the effect of having unequal sample numbers and the adverse bias this may encumber on predictions across the various species.

### Epigenome wide association studies (EWAS)

We performed a two-step meta-analysis for an epigenome-wide association study (EWAS) of age across approximately 37,000 CpGs from a mammalian array. Study samples were stratified by species and tissue type (blood or skin), and individual EWAS analyses were conducted for each stratum, provided the sample size was ≥ 15. This resulted in 11 species-tissue groups (four from blood samples and seven from skin samples) for analysis. In the first step, EWAS was performed separately for each species-tissue stratum, with associations assessed using Pearson correlation. For species with samples from both blood and skin tissues, we combined the results using the unweighted Stouffer’s method in R to generate species-level EWAS results, yielding species-specific estimates.

In the second step, we applied the unweighted Stouffer’s method to combine the species-specific EWAS results, generating a meta-analysis estimate for each CpG across all species. Pearson correlation was calculated using the ‘standardScreeningNumericTrait’ function in the WGCNA package in R, and Stouffer’s method was implemented in R.

For the blood EWAS meta-analysis, we combined the results from the four species-blood EWAS (Beluga, Pacific white-sided dolphin, Killer whale, and Bottlenose dolphin) using the unweighted Stouffer’s method to produce a meta-analysis estimate for the association with age. Similarly, for the skin EWAS meta-analysis, results were combined across seven species-skin EWAS (Bowhead whale, Beluga, Pacific white-sided dolphin, Humpback whale, Killer whale, Indo-Pacific bottlenose dolphin, and Bottlenose dolphin). In total, EWAS of age was performed on 915 samples, including 263 blood samples and 652 skin samples.

We also conducted a two-step meta-analysis for EWAS of sex-associated CpGs. In the first step, a multivariate linear regression analysis was performed for each species-tissue group. The regression model was specified as lm(CpG ~ Female + Age) as implemented in R, where CpG represents methylation levels, “Female” is an indicator variable for sex (female), and the model is adjusted for chronological age. In the second step, species-specific EWAS results were combined using the unweighted Stouffer’s method, producing a meta-analysis estimate for the EWAS of sex.

### Annotations for age-associated CpGs in cetaceans

Similar to our previous mammalian aging study^2^, we annotated age-related CpGs using chromatin state annotation, functional and biological pathway annotation through GREAT analysis, and transcription factor (TF) enrichment analysis. Importantly, we also examined the overlap with polycomb repressive complex (PRC) binding regions. The annotations were based on the top 1000 CpGs exhibiting positive correlations with age and the top 1000 CpGs showing negative correlations, as identified in our EWAS.

### Polycomb repressive complex annotation

In this study, we defined Polycomb repressive complex (PRC) annotations based on the binding of at least two transcription factor members from either PRC1 (RING1, RNF2, BMI1) or PRC2 (EED, SUZ12, EZH2) using ChIP-seq data from 49 datasets available in the ENCODE database, as previously described^2^. In total, 640 CpGs were identified in regions bound by PRC1, and 5287 CpGs were identified in PRC2-bound regions on the array.

We performed a one-sided hypergeometric test to assess the enrichment (fold change [FC] > 1) or depletion (FC < 1) of PRC-bound regions among age-related CpGs.

### Universal chromatin state annotation

To understand the chromatin context of age-related CpG sites, we utilized a comprehensive universal chromatin state annotation of the human genome. This annotation is derived from 1032 experiments mapping 32 chromatin marks across over 100 human cell and tissue types^12^. A “stacked modeling” strategy combined with a hidden Markov model (HMM) led to the identification of 100 distinct chromatin states, which were categorized into 16 major groups within the human genome.

We previously used the same chromatin state enrichment approach in our pan mammalian aging studies^2^. To evaluate the enrichment or depletion of chromatin states among age-related CpG sites, we conducted a one-sided hypergeometric test—considering a FC greater than 1 as enrichment and an FC less than 1 as depletion.

### GREAT functional enrichment analysis

We utilized the Genomic Regions Enrichment of Annotations Tool (GREAT) to annotate the potential functions of cis-regulatory regions associated with age-related CpG sites^15^. Our goal was to identify biological processes and pathways potentially linked to the top positively and negatively age-related CpG sites. To generate enrichment P values unaffected by gene size or the number of CpG sites within a gene, we carefully designed the foreground/background test. To avoid bias from array design, we used all 37,000 CpG sites from the mammalian arrays as the background in our hypergeometric enrichment test, with the genomic regions of the top 1000 age-associated CpG sites serving as the foreground. The GREAT analysis was performed with the following settings: Genome assembly: hg19, Proximal region: 5.0 kb upstream and 1.0 kb downstream of the transcription start site, Distal region: Up to 50 kb from the gene. Our analysis encompassed various databases, including Gene Ontology (GO) terms, MSigDB, Human Phenotype (including Disease Ontology), PANTHER, KEGG pathways, and Mouse Phenotype. We report gene sets that meet the criteria of having at least three overlapping genes and unadjusted hypergeometric P values less than 0.001.

### Transcription factor binding enrichment analysis

To annotate transcription factor (TF) binding enrichment in cetacean age-associated CpGs, we analyzed TFs from various families, including ATF-4-related factors, bHLH-ZIP, FOX::Etx-related, HOX::Ets-related, and others, using data from the JASPAR database (https://jaspar.elixir.no/enrichment/). Only TF binding sites identified through ChIP-seq, SELEX, or HT-SELEX methods were considered. The analysis was performed using the LOLA package in R, with background bed files based on the 37 k mammalian CpGs and test bed files based on the top 1000 age-associated CpGs showing positive and negative correlations, respectively. A buffer region of 200 bp around the CpGs was applied.

We restricted the analysis to TFs whose binding sites, when divided by background sites, were ≤ 0.5, as well as the four Yamanaka factors (OCT4, SOX2, KLF4, and MYC). This yielded a total of 274 TF genes remaining in our analysis. For TFs with multiple results generated from different ChIP-seq or other datasets, we reported the result with the median enrichment P value.

### Genome annotation

CpG annotation was performed as previously described^1,23^. The manifest file for the mammalian array and the genome annotations of the CpGs are available on Zenodo/GitHub (10.5281/zenodo.7574747). Of the CpGs on the mammalian array, 20,150 map to cetacean species’ genomes^1^.

## Supplementary Information

Supplementary Information 1.

Supplementary Information 2.

Supplementary Information 3.

Supplementary Information 4.

Supplementary Information 5.

Supplementary Information 6.

## Data Availability

The data will be made publicly available on Gene Expression Omnibus as part of the data release from the Mammalian Methylation Consortium. The manifest file of the mammalian array and genome annotations of the CpGs can be found on GitHub (10.5281/zenodo.7574747) https://github.com/shorvath/MammalianMethylationConsortium. The mammalian methylation array is broadly available to the research community from the non-profit Epigenetic Clock Development Foundation (https://clockfoundation.org/).
