# Supplementary material for: Enhancing epigenetic aging clocks in cetaceans: accurate age estimations in small endangered delphinids, killer whales, pilot whales, belugas, humpbacks, and bowhead whales
Source: Sci Rep. 2025 Feb 3;15:4048. doi: 10.1038/s41598-025-86705-5 (PMC11791194; doi:10.1038/s41598-025-86705-5)
Supplement: Supplementary file 1 — Supplementary Information 1. [file 41598_2025_86705_MOESM1_ESM.pdf]

Supplementary Material

Title: Enhancing Epigenetic Aging Clocks in Cetaceans: Accurate Age Estimations in small endangered Delphinids, killer whales, Pilot Whales, Belugas, Humpbacks, and Bowhead whales.

Joseph A. Zoller, Ake T. Lu, Amin Haghani, Steve Horvath, Todd Robeck

Supplementary Table 1. Mean age (male and female) at sexual maturity and gestation time in years for each respective species used in epigenetic clock development.

**Species common name (S*cientific Latin name*)**	**Age at Sexual Maturity**	**Gestation Time**
Beluga (*Delphinapterus leucas*)	11.0	1.28
Bowhead whale (*Balaena mysticetus*)	22.5	1.16
Commerson's dolphin (*Cephalorhynchus commersonii*)	5.5	0.96
Common bottlenose dolphin (*Tursiops truncatus*)	8.93	1.03
Harbor porpoise (*Phocoena phocoena*)	3.45	0.88
Humpback whale (*Megaptera novaeangliae*)	7.96	0.94
Indo-Pacific bottlenose dolphin (*Tursiops aduncus*)	6.75	1.00
Killer whale (*Orcinus orca*)	10.5	1.46
Pacific white-sided dolphin (*Lagenorhynchus obliquidens*)	7.0	0.98
Rough-toothed dolphin (*Steno bredanensis*)	10.0	1.03
Short-beaked common dolphin (*Delphinus delphis*)	4.57	0.96
Short-finned pilot whale (*Globicephala macrorhynchus*)	12.06	1.24

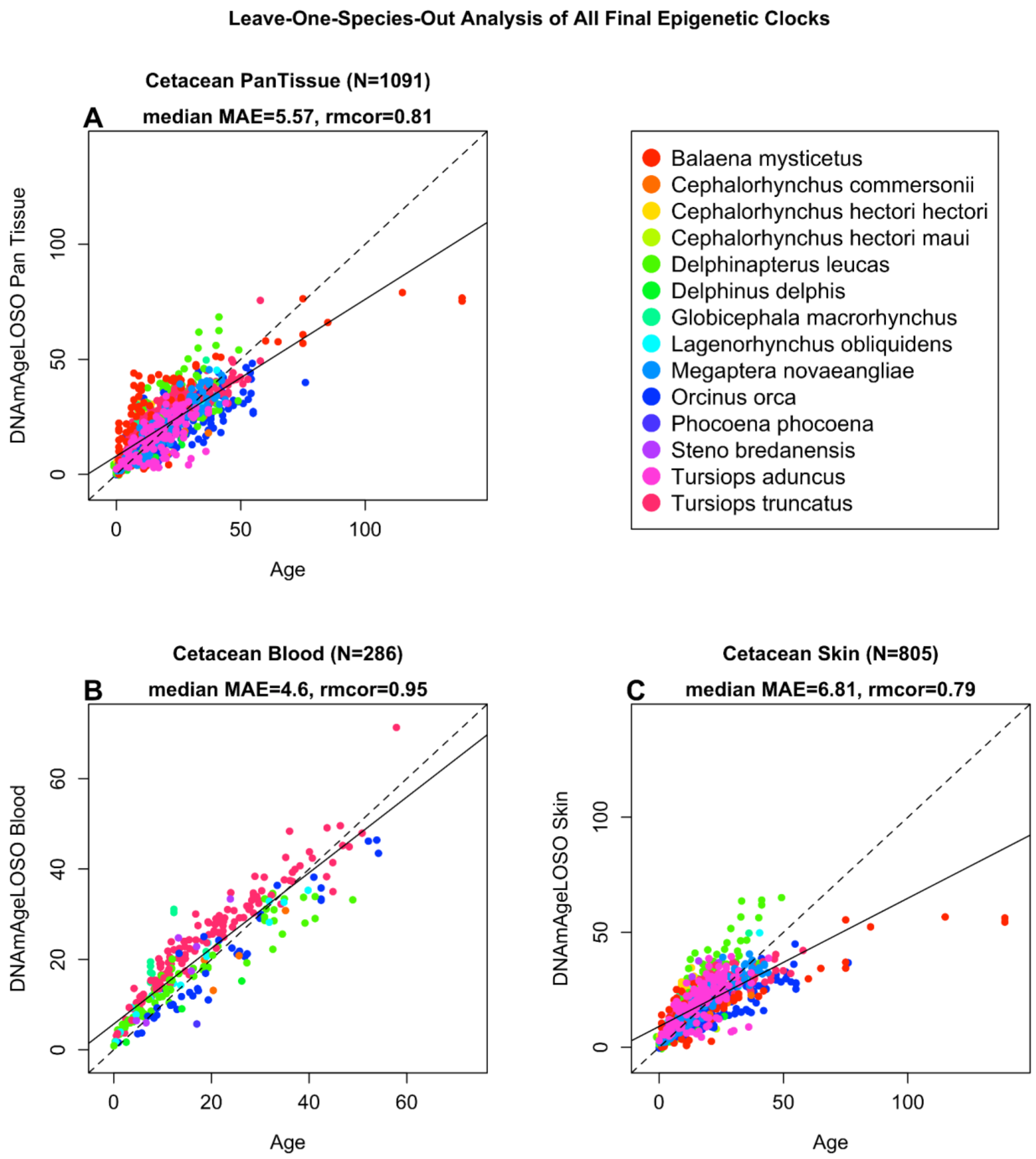

Supplementary Fig. 1. Leave One Species Out Cross Validation (LOSOCV) study of the epigenetic clock for cetaceans. Each dot corresponds to a tissue sample from cetaceans. Dots are colored by species (see legend in figure). Panels (A-C) represent cross validations of different tissue strata clocks, with blood and skin combined (A), blood only (B) and skin only (C). The LOSOCV estimates lend themselves for estimating the performance of these multi-species cetacean clocks in any cetacean species that were not part of the training set. Each panel depicts a linear regression fitted line (black solid line), a diagonal identity line (y = x [black dashed line]), the sample size (N), repeated measures correlation (rmcor) across all samples, and median value of the median absolute error (MAE) across species.

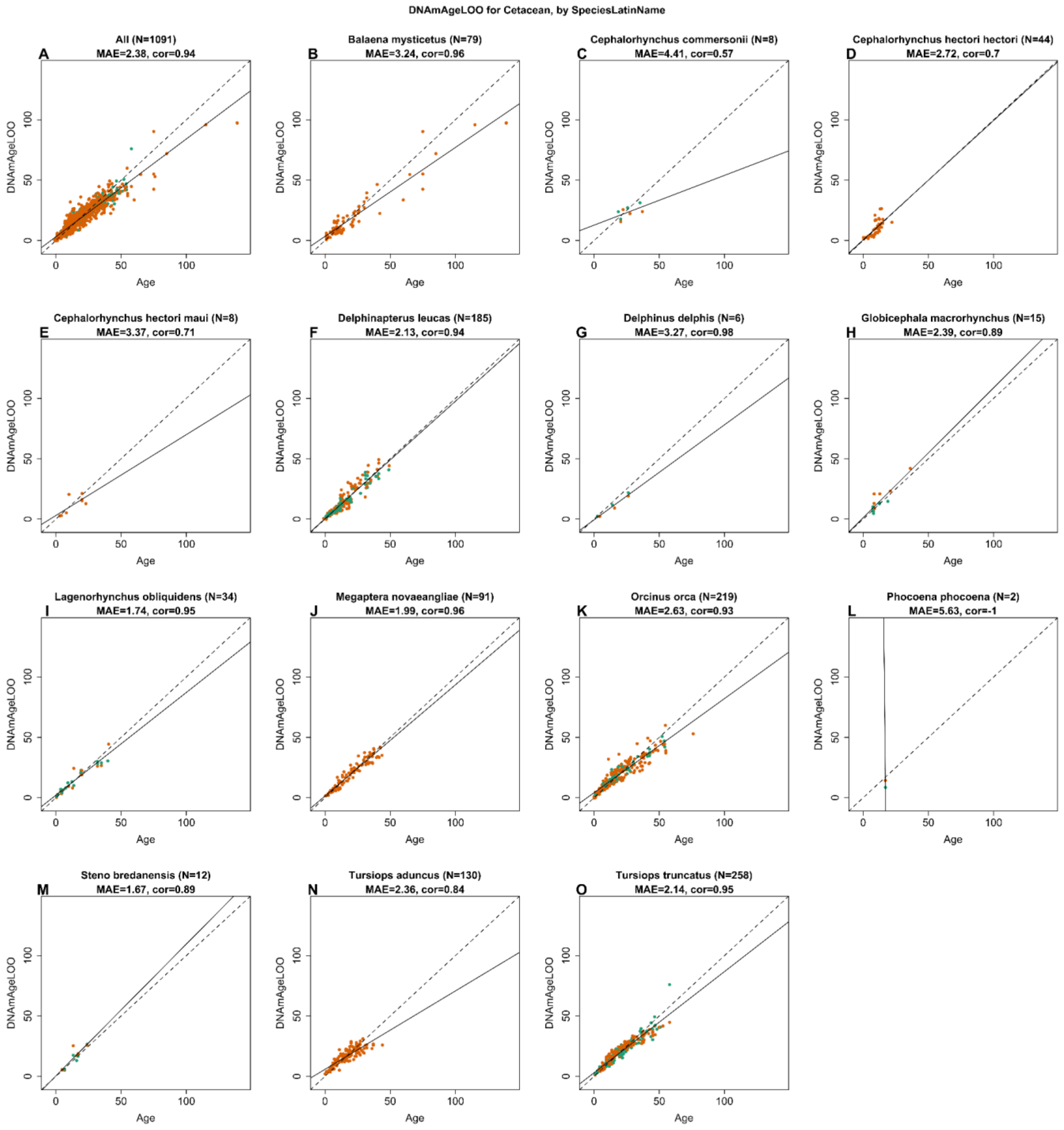

Supplementary Fig. 2. Leave One Out Cross Validation (LOOCV) study of the epigenetic clock for cetacean blood and skin. Each dot corresponds to a tissue sample from cetaceans. Dots are colored by tissue type (teal = blood, orange = skin). Panels (A-M) represent cross validation of the multi-species, multi-tissue cetacean clock, showing all samples combined (A), and showing individual species (B-M). Each panel depicts a linear regression fitted line (black solid line), a diagonal identity line (y = x [black dashed line]), the sample size (N), Pearson's correlation (cor), and median absolute error (MAE).

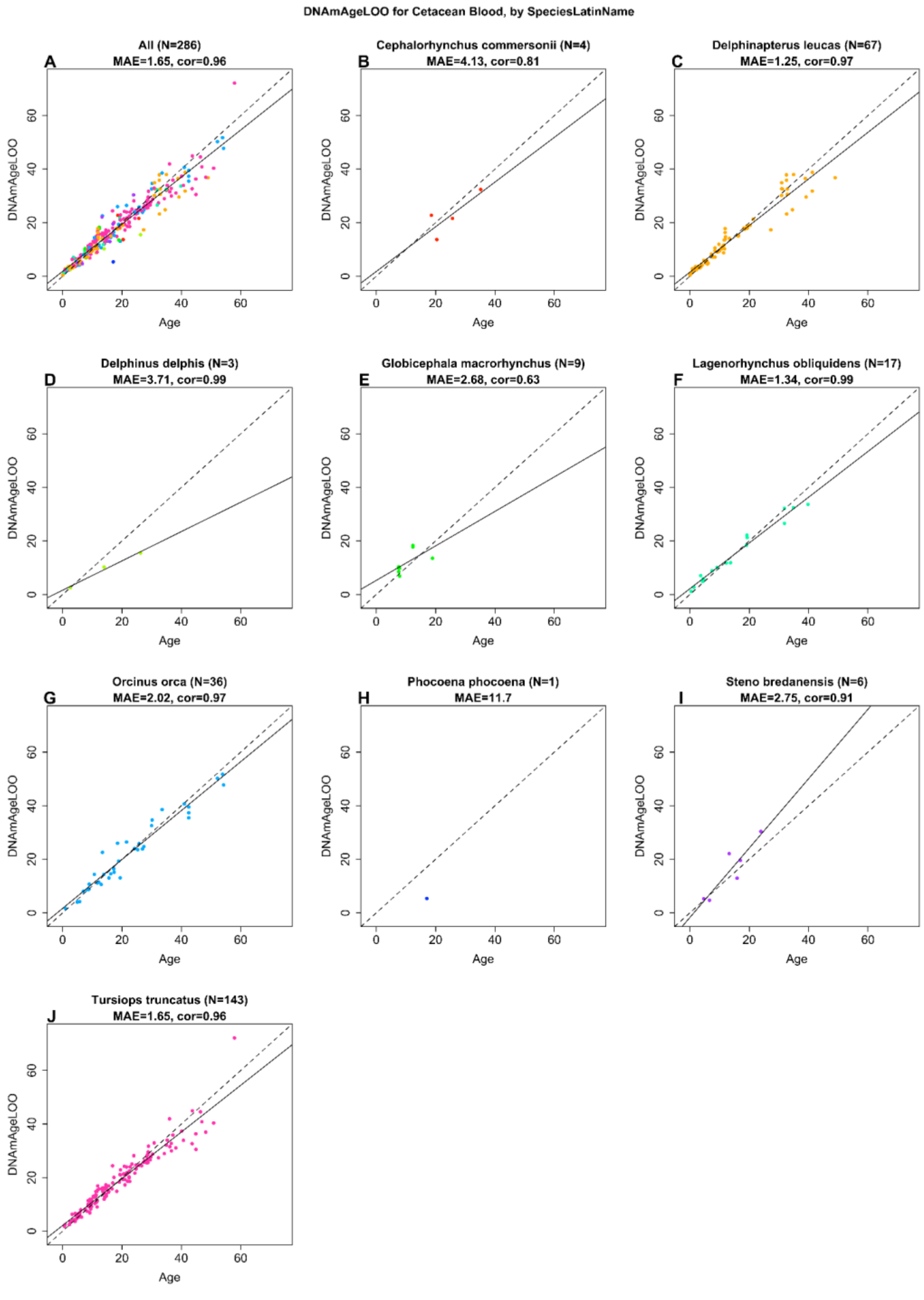

Supplementary Fig. 3. Leave One Out Cross Validation (LOOCV) study of the epigenetic clock for cetacean blood only. Each dot corresponds to a tissue sample from cetaceans. Dots are colored by species. Panels (A-J) represent cross validation of the multi-species, blood-only cetacean clock, showing all samples combined (A), and showing individual species (B-J). Each panel depicts a linear regression fitted line (black solid line), a diagonal identity line (y = x [black dashed line]), the sample size (N), Pearson's correlation (cor), and median absolute error (MAE).

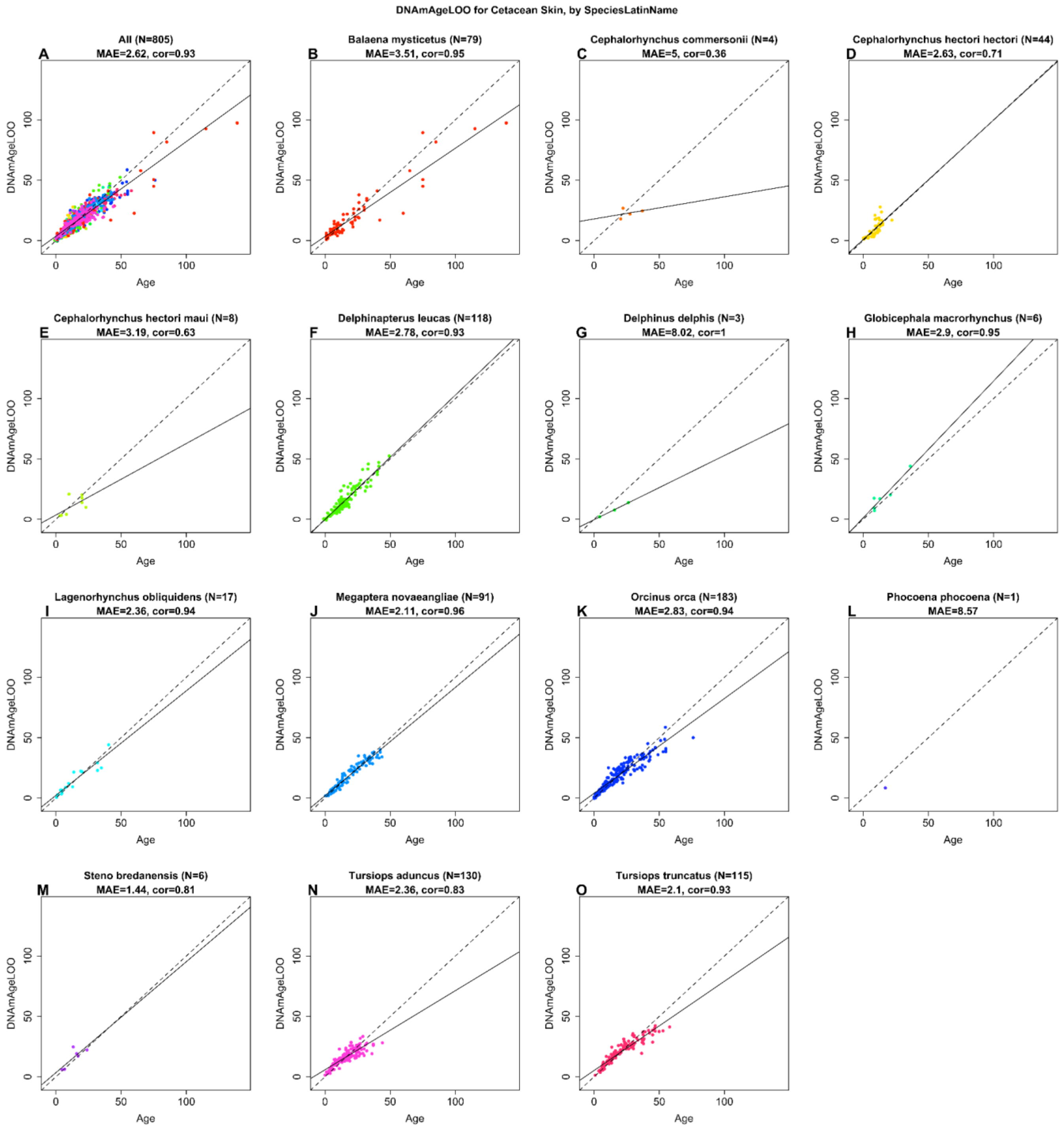

Supplementary Fig. 4. Leave One Out Cross Validation (LOOCV) study of the epigenetic clock for cetacean skin only. Each dot corresponds to a tissue sample from cetaceans. Dots are colored by species. Panels (A-M) represent cross validation of the multi-species, skin-only cetacean clock, showing all samples combined (A), and showing individual species (B-M). Each panel depicts a linear regression fitted line (black solid line), a diagonal identity line (y = x [black dashed line]), the sample size (N), Pearson's correlation (cor), and median absolute error (MAE).

General description of age transformation

The cetacean clocks all used a log linear transformation that is similar to those employed for the human pan-tissue clock (Horvath 2013).

An elastic net regression model (implemented in the glmnet R function) was used to regress a transformed version of age on the beta values in the training data. The glmnet function requires the user to specify two parameters (alpha and beta). Since I used an elastic net predictor, alpha was set to 0.5. But the lambda value of was chosen by applying a 10-fold cross validation to the training data (via the R function cv.glmnet).

The elastic net regression results in a linear regression model whose coefficients b_0, b_1, . . . , relate to transformed age as follows

F(chronological age)=$b_0+b_1CpG_1+ \ldots +b_pCpG_p$+error

Note that the intercept term is denoted by b_0. The coefficient values can be found in Supplementary Table. Based on the coefficient values from the regression model, DNAmAge is estimated as follows

$DNAm$Age=$F^{-1}(b_0+b_1CpG_1+ \ldots +b_pCpG_p)$,

where $F^{-1}(y)$ denotes the mathematical inverse of the function F(.). Thus, the regression model can be used to predict to transformed age value by simply plugging the beta values of the selected CpGs into the formula.

Defining Properties of the log linear transformation

As indicated by its name, the "log-linear" function, has a logarithmic dependence on age before the average age of sexual maturity (of the species) and a linear dependence after Age at Sexual Maturity (of the species). The parameters necessary for this transformation are provided in Supplementary Table 1.

We used a piecewise transformation parameterized by Age of Sexual Maturity (A) and Gestational Period in years (G).

The transformation is F(x), given by

$$F(x) = g\left(\frac{x+G}{1.5*A+G}\right) \text{ where } g(t) = \begin{cases} \log(t), & for\ t \leq 1 \\ t-1, & for\ 1 \leq t \end{cases}$$

Explicitly, F(x) is given by

$$F(x) = \begin{cases} \log\left(\frac{x+G}{1.5*A+G}\right), & for\ 0 \leq x \leq 1.5*A \\ \frac{x-1.5*A}{1.5*A+G}, & for\ 1.5*A \leq x \end{cases}$$

In order to use this transformation to predict Age on *new samples*, you will need to use the *inverse* transformation, $F^{-1}(y)$, given by

$$F^{-1}(y) = \begin{cases} (1.5*A+G)*\exp(y)-G, & for\ y \leq 0 \\ (1.5*A+G)*y+1.5*A, & for\ y \geq 0 \end{cases}$$

For predicting age, you will apply the inverse transformation to coefficient-weighted sum. That is,

$$DNAmAge = F^{-1}(x*\beta)$$

where β is the vector of coefficients and x is the vector of methylation values, with an intercept term.
